# Acupuncture as adjuvant therapy for diabetic foot: A protocol for systematic review

**DOI:** 10.1097/MD.0000000000019502

**Published:** 2020-03-20

**Authors:** Maosheng Lee, Huilin Li, Deliang Liu

**Affiliations:** a Guangzhou University of Chinese Medicine, University Town of Guangzhou, Panyu District, Guangzhou City; b Department of Endocrinology, Shenzhen Traditional Chinese Medicine Hospital, Futian District, Shenzhen City, Guangdong Province, China.

**Keywords:** acupuncture, diabetic foot, adjuvant therapy, systematic review, meta-analysis

## Abstract

**Background:**

Diabetic foot (DF) problems are common throughout the world, about one-fourth of them develop a foot ulcer and serious cases would suffer from amputation, which seriously affects the patient's work and life. Previous studies indicated that acupuncture as adjuvant therapy would be effective in treating DF. However, these studies have no consistent results. Therefore, the aim of our study was to explore the efficacy and safety of acupuncture as adjuvant therapy for DF.

**Methods:**

The randomized controlled trials associated with acupuncture therapy (or as adjuvant therapy) for DF will be included. We will search 6 electronic databases relevant to health sciences, including PubMed, Embase, the Cochrane Library, the Chinese databases Sino-Med, CNKI, and WANFANG database. All searches were from databases inception to March 30, 2019. The primary outcomes are the total curative effective rate, and the hemodynamic parameter and adverse events will be deemed as secondary outcomes. The Stata15.1 software and Review Manager (RevMan 5.3; Cochrane Collaboration, Copenhagen, Denmark) will be used for analysis, to assess the bias risk, subgroup analysis, and data synthesis.

**Results:**

In this systematic review and meta-analysis, we will synthesize the studies to assess the safety and efficacy of acupuncture as adjuvant therapy for DF.

**Conclusion:**

The summary of our study will clarify whether acupuncture as adjuvant therapy could be an efficient method for DF.

## 1 Introduction

Diabetic foot (DF), is known as a complication of diabetes mellitus, which is common throughout the world and the lifetime risk of a patient with diabetes mellitus develops a foot ulcer would be high as 25%.^[1]^ A quarter of serious cases might even lead to amputation,^[2]^ and a lower limb is lost somewhere in the world as a consequence of diabetes mellitus every 30 seconds.^[3]^ It can seriously reduce the patients’ quality of life and affects social participation.^[4]^ DF ulceration and amputations were estimated to cost US healthcare payers $10.9 billion in 2001.^[5,6]^

The perennial hyperglycemia contributes to the development of peripheral arterial diseases and neuropathy,^[7,8]^ which combination might lead to foot ulcers. To prevent and treat the DF, except for hypoglycemic treatment, most patients were probably expecting to promoting vascular surgery or modern medications.^[9]^ The general treatment options on DF include glycemic control, extensive local wound care, and prompt revascularization.^[10]^ However, the poor healing rate is still a fatal cause leading to low quality of life and enormous medical expenditures.^[11]^

Acupuncture, a critical component of traditional Chinese medicine, has a history of thousands of years in China and widely uses to treat many diseases, including vascular diseases. Acupuncture refers to insert the needless into the specific acupoints on body for facilitating recovery, which is commonly used to treat diabetic neuropathy^[12,13]^ and DF.^[14]^ Up to now, there are numerous of studies about acupuncture therapy (or as adjuvant therapy) for DF; however, these results from different studies are still inconclusive. Although abundant clinical researches exist about acupuncture as adjuvant therapy for intervention in DF, a systematic evaluation and meta-analysis about its safety and efficacy still remains insufficient. Therefore, our study aim to synthesize the randomized controlled trials (RCTs) and critically access the comparative effectiveness and safety of acupuncture as adjuvant therapy intervention in clinical trials, anticipating to aiding the advanced treatment of DF.

## 2 Methods

### 2.1 Study registration

This protocol of this study has been registered on the International Prospective Register of Systematic Reviews (PROSPERO), the registration number is CRD42018086548. The protocol will be conducted severely under the guideline of Preferred Reporting Items for Systematic Reviews and Meta-analyses Protocols (PRISMA-P).^[15]^

### 2.2 Eligibility criteria

#### 2.2.1 Type of study

Only RCTs were included in the present review, such as traditional acupuncture, warm-needle techniques, and electroacupuncture. No publication date and no language restrictions were imposed on the initial search.

#### 2.2.2 Type of participant

The patients, aged 18 years or older, suffering from DF will be included, regardless of the limitation of gender and nationality. The Wagner grade evaluation levels 0 to 4 are included.

#### 2.2.3 Type of intervention

We will include studies in which intervention group applying acupuncture alone or in combination with other forms of treatment as the intervention, such as Chinese herbal medicine and western medicine, the control group undergoing drug treatment and not undergoing any acupuncture (electroacupuncture, warm acupuncture, auricular acupuncture, scalp acupuncture, dry needling, acupoint injection, press needle, acupressure, acupoint catgut embedding, etc) intervention.

#### 2.2.4 Types of outcome measurements

##### 2.2.4.1 Primary outcome

The primary outcome is the total curative effective rate.

##### 2.2.4.2 Secondary outcomes

Secondary outcomes include the hemodynamic parameter and side effects caused by acupuncture. In the subgroup analysis, the treatment times to DF healing and the therapeutic method that acupuncture combined with other treatments would be expounded.

#### 2.2.5 Exclusion criteria

The studies with the following situation will be excluded: the participants were diagnosed with the unclear diagnostic criteria; duplicated data or the data cannot be extracted. Observational studies, retrospective studies, nonrandomized trials, quasiexperimental studies, and animal studies were excluded. Additionally, the studies with in-sufficient data or lacking effective sort were also not included.

### 2.3 Search methods for identification of studies

#### 2.3.1 Electronic data sources

Six electronic databases from inception to March 2019 will be searched: PubMed, Embase, the Cochrane Library, and the Chinese databases Sino-Med, Chinese National Knowledge Infrastructure (CNKI), and Wanfang database.

#### 2.3.2 Other resources

Other resources of related studies will be searched. The PROSPERO Register of Controlled Trials, the Cochrane Central Register of Controlled Trials, and the Cochrane Complementary Medicine Field Specialized Register were also retrieved. Relevant conference papers or other relevant literatures were also conducted.

### 2.4 Search strategy

We formulated search strategies for each database used the keywords such as acupuncture, DF, and RCT. Subsequent databases searches used MeSH headings, including acupuncture and DF, in addition to keywords from the initial retrieval. And other article searches were conducted by reviewing the literature lists of relevant research articles. Used PubMed as an example, the search strategy for PubMed is summarized in Table 1. Additionally, we will make appropriate modifications in accordance with the actual requirements.

**Table 1:**
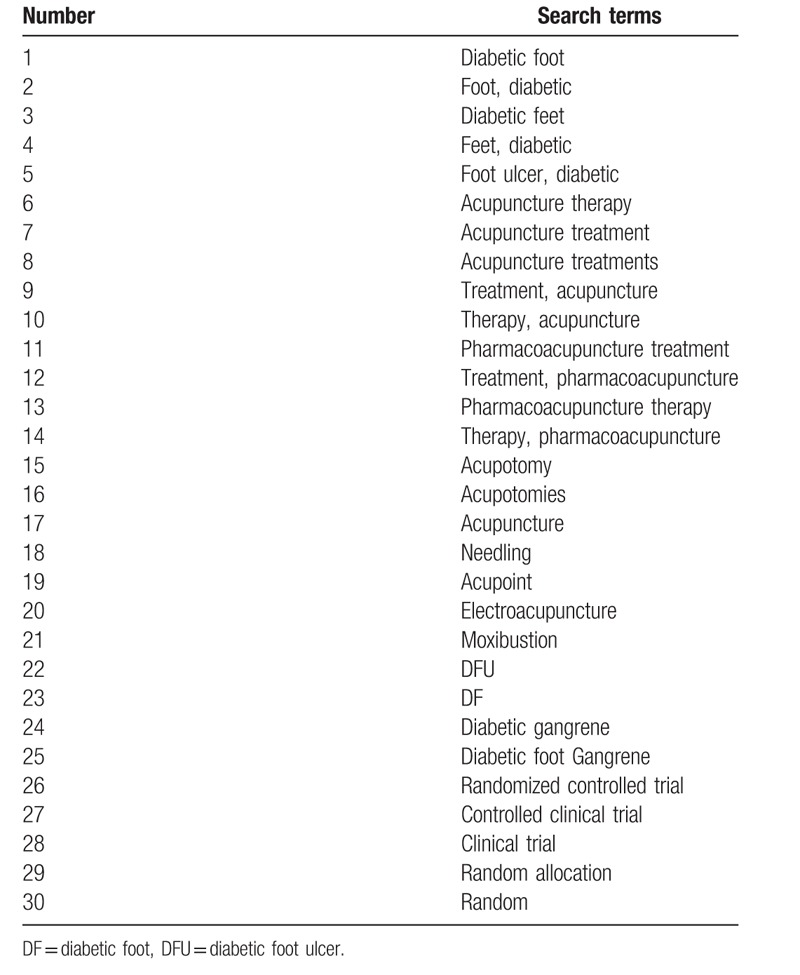
Search strategy for PubMed.

### 2.5 Data extraction

#### 2.5.1 Selection of studies

**Figure 1:**
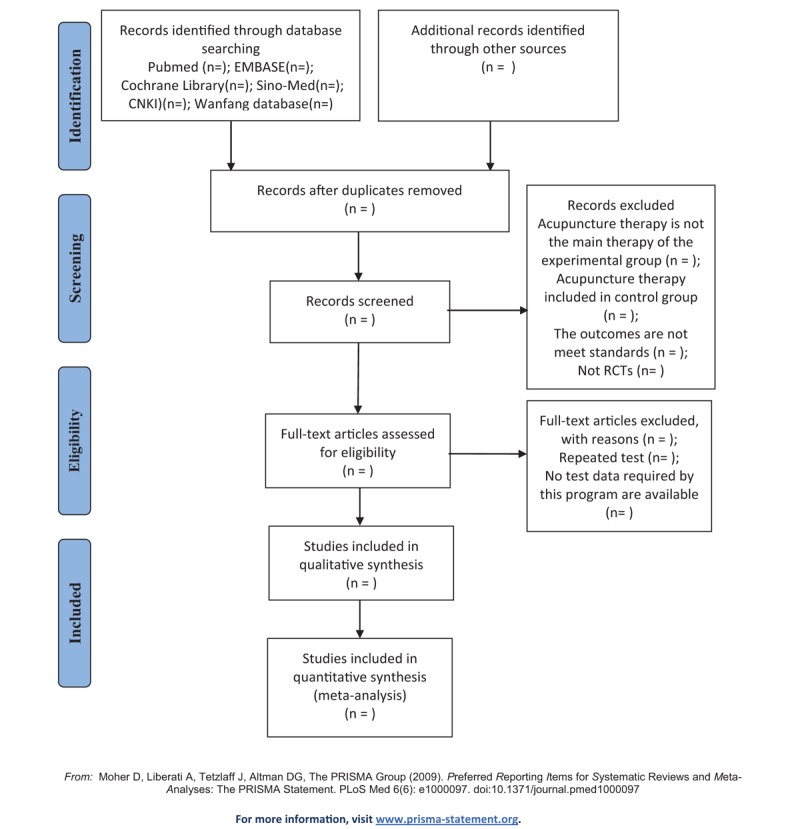
Flow diagram of studies identified. CNKI = Chinese National Knowledge Infrastructure, RCTs = randomized controlled trials, Sino-Med = the Chinese databases Sino-Med.

Two researchers will independently obtain the studies from the databases mentioned earlier and access the titles and abstracts of each study, and then exclude the obviously unqualified literature. Later, they will strictly screen the studies by following the eligibility criteria and exclusion criteria. The different opinions will be resolved by discussions. The final selection procedure is indicated in Figure 1 abide by the PRISMA guidelines.^[16]^

#### 2.5.2 Data extraction and management

Retrievals were actualized and data extracted by 2 independent investigators (HLL and MSL). Each study was evaluated for design, participants’ characteristics, interventions, eligibility criteria, outcomes measures and research quality, and detail recorded in an Excel file.

#### 2.5.3 Assessment of risk of bias in included studies

The 2 reviewers will independently use the bias tool of Cochrane Handbook for Systematic Reviews of Interventions^[17]^ to evaluate the risk of bias of the final included studies. The evaluated items include random sequence generation, allocation concealment, blinding to participants, personnel and outcome, incomplete outcome data, selective reporting, and other biases. The quality of the studies will be divided into 3 levels: “low risk of bias,” “high risk of bias,” and “unclear risk of bias.”

#### 2.5.4 Measures of treatment effect

For the dichotomous variables outcomes, the total effective rate and adverse events, we will analyze the rate ratio. The mean difference will be used to evaluate the continuous variables data. The 95% confidence interval will be presented for both dichotomous outcomes and continuous outcomes.

#### 2.5.5 Management of missing data

We will contact the original author for the missing or incomplete data. The incomplete data will be dislodged if it cannot be supplemented.

#### 2.5.6 Assessment of heterogeneity

Heterogeneity will be assessed by the Cochran *Q* statistic and quantified by the *I*^2^ statistic. If *I*^2^ *>* 50%, the studies were considered to be heterogeneity, a random-effects models would be used. If *I*^2^ *<* 50%, a fixed-effects models was implemented. *I*^2^ (25–50%) as moderate level heterogeneity.

#### 2.5.7 Assessment of reporting biases

We will apply the funnel plots to evaluate the reporting biases if the included studies are more than ten trials. Otherwise, the STATA13.0 software will be conducted to perform the Egger test.

#### 2.5.8 Subgroup analysis

If there are adequate studies (>10), we will conduct a subgroup analysis to explore the different of treatment time to DF healing and the different therapeutic method that acupuncture combined with other treatments would be expounded.

#### 2.5.9 Sensitivity analysis

We will carry out the sensitivity analysis to access the quality and robustness when the significant statistical heterogeneity arose according to sample size and insufficient data.

### 2.6 Grading the quality of evidence

The Grading of Recommendations Assessment, Development, and Evaluation (GRADE) guidelines^[18]^ method will be applied to evaluate the quality of evidence of the pooled trials from five aspects, included limitation of study design, inconsistency, indirectness, imprecision, and bias of publication. Additionally, the levels of evidence quality will be classified into 4 levels: high, moderate, low, and very low.

### 2.7 Ethics and dissemination

The ethics approval is not needed as the data are extracted from the published literature and they are not related to the individual patient's data. This systematic review and meta-analysis will be published in a peer-reviewed journal.

## 3 Discussion

The prevalence of DF increases year by year with the increase of the morbidity rate of diabetes mellitus, which brings great influence and psychological burden to the life and work of patients. However, the effective treatment for diabetic is still controversial. Although controlling blood sugar is the most basic way, many patients have difficulty maintaining that blood sugar is controlled in a relatively stable state at the later period DF. To this end, they are suffering from sharp pain and facing with the amputation. For the patients with DF, who characterized by thickened vascular and arterial stenosis, the lower extremity arterial blood flow has obvious changes.^[19]^

Acupuncture therapy (or as adjuvant therapy) may be an efficacy and safety treatment for DF, which may work through improving vascular circulation in the lower extremities or regulating immunity to relieve pain and promote the wound heals. However, the evidences of efficacy and safety of acupuncture as adjuvant therapy for DF is insufficient and the potential mechanisms leave several unknown. Therefore, a systematic review and meta-analysis of recent literatures is needed to access the clinical efficacy and safety of acupuncture as adjuvant therapy for DF. For further meaning, the results will provide the reliable evidence for the clinicians and patients in the treatment of DF.

## Author contributions

**Conceptualization:** Maosheng Lee.

**Methodology:** Huilin Li, Maosheng Lee, Deliang Liu.

**Supervision:** Huilin Li.

**Writing – original draft:** Maosheng Lee, Deliang Liu.

**Writing – review & editing:** Maosheng Lee, Huilin Li.
